# A Pharmacist-Led Point-of-Care INR Clinic: Optimizing Care in a Family Health Team Setting

**DOI:** 10.1155/2013/691454

**Published:** 2013-12-12

**Authors:** Jennifer Rossiter, Gursharan Soor, Deanna Telner, Babak Aliarzadeh, Jennifer Lake

**Affiliations:** ^1^Department of Family and Community Medicine, University of Toronto, Toronto, Ontario, Canada; ^2^South East Toronto Family Health Team, Toronto, Ontario, Canada; ^3^Department of Family Medicine and the Department of Emergency Medicine at Headwaters Health Care Centre, Orangeville, Ontario, Canada; ^4^North Toronto Research Network, Toronto, Ontario, Canada; ^5^Leslie Dan Faculty of Pharmacy and the Department of Family and Community Medicine, University of Toronto, Toronto, Ontario, Canada

## Abstract

*Purpose*. Monitoring patients' international normalized ratio (INR) within a family medicine setting can be challenging. Novel methods of doing this effectively and in a timely manner are important for patient care. The purpose of this study was to determine the effectiveness of a pharmacist-led point-of-care (POC) INR clinic. *Methods*. At a community-based academic Family Health Team in Toronto, Canada, charts of patients with atrial fibrillation managed by a pharmacist with usual care (bloodtesting at lab and pharmacist follow up of INR by phone) from February 2008 to April 2008 were compared with charts of patients attending a weekly POC INR clinic from February 2010 to April 2010. Time in therapeutic range (TTR) was measured for both groups. *Results*. 119 patient charts were reviewed and 114 had TTR calculated. After excluding patients with planned inconsistent Coumadin use (20), such as initiating Coumadin treatment or stopping for a surgical procedure, the mean TTR increased from 64.41% to 77.09% with the implementation of the POC clinic. This was a statistically significant difference of 12.68% (CI: 1.18, 24.18; *P* = 0.03). *Conclusion*. A pharmacist-led POC-INR clinic improves control of anticoagulation therapy in patients receiving warfarin and should be considered for implementation in other family medicine settings.

## 1. Introduction

Atrial fibrillation is the most common cardiac dysrhythmia, with a prevalence cited in the literature of 10% in individuals over the age of 80 [1, 2]. Fibrillatory conduction of the atria results in blood stasis, formation of thrombotic clots, and an increased risk of thromboembolic events, such as stroke. Studies have shown that oral anticoagulant therapy, such as warfarin, can help reduce the risk of thromboembolic events by up to 60% compared to those receiving no treatment [3]. Warfarin has a narrow therapeutic range and can also be associated with hemorrhagic side effects, including a known increased risk of gastrointestinal bleeding and hemorrhagic stroke [4]. Regular monitoring of patients on warfarin to maintain levels within therapeutic range is necessary to decrease the incidence of such side effects.

This need for continuous monitoring, along with recommendations for an increasing number of patients on warfarin therapy, has led to the development of alternative models for monitoring oral anticoagulant therapy. Traditionally, oral anticoagulation therapy has been monitored by specialists or directly by family physicians, as was the case at the South East Toronto Family Health Team (SETFHT), a community based academic teaching unit. With the development of multidisciplinary Family Health Teams (FHTs) in Ontario, Canada, expanding roles for nonphysician health care providers in patient care have occurred. A recent study found nurse-led INR monitoring of INR to be equally effective as physician monitoring [5]. In July 2007, SETFHT changed its delivery of oral anticoagulation monitoring from individual physician based to a centralized model in which a pharmacist followed the blood testing results and managed all patients in the clinic who were taking warfarin. In May 2008, the model was changed to a weekly pharmacist-led point-of-care (POC) INR clinic, where patients would come for an appointment with the pharmacist, have a POC INR done on site using the CoaguCheck XS Machine (Roche Diagnostics), and warfarin dose adjusted immediately based on these results.

The objective of this study was to determine the efficacy of the pharmacist-led POC INR clinic compared with routine pharmacist managed INR.

## 2. Methods

A chart audit using quasiexperimental study was performed by searching electronic charts at SETFHT to identify patients who were receiving warfarin therapy. Patients were included if they were taking warfarin for atrial fibrillation or atrial flutter, and if they had a target INR of 2 to 3. Baseline data was collected on all patients who were taking warfarin from Feb 1, 2008 to April 30, 2008. This timeline was chosen as it was just prior to starting the POC INR clinic and was 9 months into the pharmacist-led monitoring of INR. Data was collected through chart review and included INR values, patient demographics, and comorbidities. Planned inconsistencies in warfarin therapy administration, such as initiating, discontinuing, or holding warfarin during the study period (for instance, if a patient was undergoing a surgical procedure), were also recorded. Similar data was also collected for patients who were monitored in the POC INR clinic from Feb 1, 2010 to April 30, 2010. This timeline was chosen as the POC INR clinic had been running for 2 years and it was thought that other external variables (holidays, seasonal vitamin K variations) would be minimized by using the same 3-month period. Sample size was based on convenience and included all patients meeting criteria. Patients were considered to be monitored in the POC clinic as long as the POC clinic was the source of at least half of their recorded INR values.

Time in therapeutic range (TTR) is an estimate of the number of days that a patient has an INR value within their target range. TTR is considered the standard for monitoring oral anticoagulation therapy in patients on warfarin [6]. TTR was determined for both groups of patients using the Rosendaal method of linear interpolation [7]. Linear mixed models were used to compare the TTRs from 2008 and 2010 to evaluate the effect of the POC clinic as this method can handle missing data without deleting subjects in a repeated measure study. Data from patients with planned inconsistent warfarin use during the study period were then excluded.

We used SAS statistical program, version 9.2, for data analysis. This study was approved by the Research Ethics Board at the Toronto East General Hospital.

## 3. Results

119 patients were identified who met the inclusion criteria (Table 1). Of these patients, 51.3% (*n* = 61) were female and mean age was 78.8 years.

**Figure 1:**
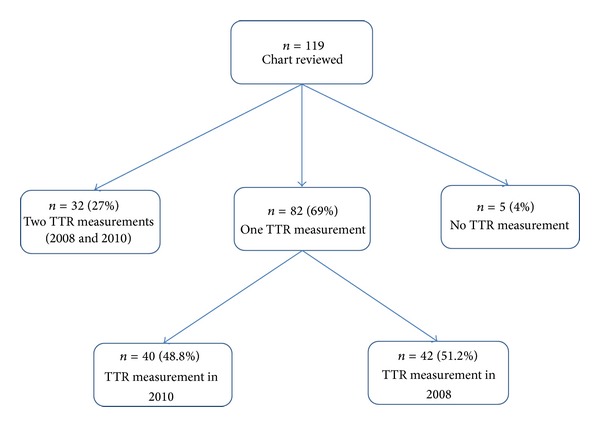
Flow diagram presenting the grouping of patients based on available TTR measurement.

Figure 1 presents the grouping of patients based on availability of estimated TTR measurement in 2008 and 2010.

Of 119 patients, 5(4%) had no estimate of TTR and 32(27%) had TTR estimate for both years. Those included in only one cohort was almost equally divided between 2008 (*n* = 42) and 2010 (*n* = 40). Distribution of study characteristics were similar, except for frequency of diabetes and dyslipidemia that is higher in the 2010 group. All 114 subjects with a calculated TTR were included in the analysis. The TTR in 2008 was compared to the TTR in 2010 to evaluate the effect of the POC clinic. The estimated marginal mean TTR for year 2008 was 64.8% and for year 2010 was 70.4%; the mean increase in TTR from 2008 to 2010 was 6.34% (95% CI −4.30, 16.99). Type III test for the effect of POC was not statistically significant (*P* = 0.24).

**Figure 2:**
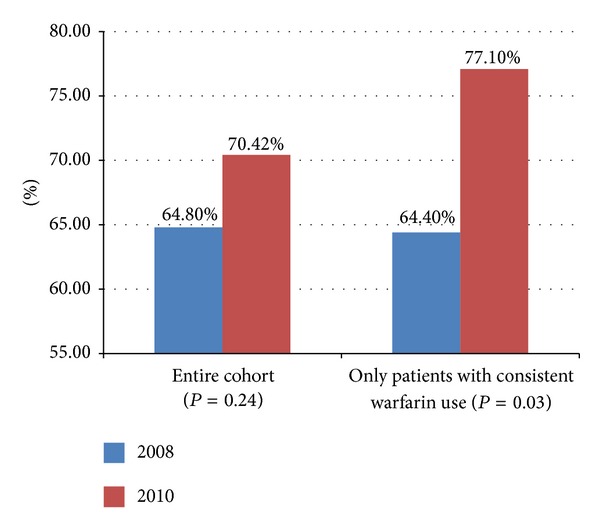
Results of TTR for entire cohort (*n* = 114) and cohort with patients excluded for planned starts/interruptions (*n* = 94).

A total of 20 patients (16.8%) had planned inconsistent warfarin usage during the study time. This included patients who were initiating or discontinuing warfarin therapy during the study period and those holding due to a procedure or medication interaction. When the patients with planned inconsistent warfarin usage were excluded from the analysis, mean TTR in 2008 was 64.4% and the mean TTR in 2010 was 77.1%. Using linear mixed models, the TTRs were on average 12.68% (CI: 1.18, 24.18) higher in 2010 than in 2008. This difference and Type III test for the effect of POC were statistically significant (*P* = 0.03). This is illustrated in Figure 2.

## 4. Discussion

Our study indicates a significant increase in TTR after the implementation of the pharmacist-led POC INR clinic. Pharmacist-managed INR has been shown to be more effective than physician-managed INR. A study in the US looked at patients whose warfarin therapy was stabilized in a pharmacist-managed anticoagulation clinic and then discharged to the care of their family physicians [8]. These patients showed a significant decrease in INR control once care was assumed by their family physicians. There have been some studies describing benefits of POC INR in managing patients on warfarin in different settings. Several descriptive case studies have highlighted the benefit of POC INR testing by pharmacists for patients who live in rural or remote areas with limited access to hospitals and medical clinics [9, 10]. A retrospective cohort study found improved INR control in patients at cardiology clinics who took part in a nurse-run, mobile POC anticoagulation therapy clinic [11]. To our knowledge, our study is the first one to examine the effectiveness of a pharmacist-led POC INR clinic within a family medicine setting. An advantage of a POC clinic is having immediate access to INR results, eliminating lag time between blood test and medication adjustment. As well, patients have the opportunity to discuss any medication concerns with the pharmacist at the time of the POC INR appointment. As patients on warfarin are at higher risk of drug interactions and adverse events, this benefit is important.

This study had several limitations. The calculation of TTR assumes that the change in INR between two known points is linear. It is not possible to take into account the natural fluctuations in INR, for example, due to dietary changes. Nevertheless, the Rosendaal method of linear interpolation is an accepted method for calculation of TTR. Missing TTR measurements for a significant number of patients was another limitation; to conduct a study with repeated measurement design pre-and post- POC implementation, availability of both measurements for all patients would have been ideal. However, this was a quasiexperimental study, and we did not have control on completeness or availability of data in patient records. A small number of patients with missing TTR in both pre- and post-POC implementation were excluded. We employed linear mixed modeling techniques to analyze the remaining data and reduced the impact of missing information

Findings of this study suggest that a centralized POC INR clinic led by a pharmacist improves control of anticoagulation therapy in patients receiving warfarin for atrial fibrillation in a family medicine setting. Given the positive results shown in our analysis, expanding the POC INR clinic to other Family Health Teams in the community is an option worthy of consideration.

## Figures and Tables

**Table 1:** Baseline characteristics of patients.

	Group by year of TTR measurement*	
	2008 (*n* = 74)	2010 (*n* = 72)
Age (mean, SD)	80.5 (9.3)	78.3 (11.1)
Female (%)	51.4	51.4
Heart failure (%)	32.4	30.6
Hypertension (%)	52.7	54.2
Dyslipidemia (%)	29.7	38.9
Diabetes (%)	23.0	29.2
Cerebrovascular Accident (%)	25.7	27.8
Gastro-Intestinal Bleed while taking Warfarin (%)	4.1	1.4

*32 patients are reported in both 2008 and 2010 cohorts.
